# Supplementary material for: Exploring the Association between Delirium and Malnutrition in COVID-19 Survivors: A Geriatric Perspective
Source: Nutrients. 2023 Nov 9;15(22):4727. doi: 10.3390/nu15224727 (PMC10674410; doi:10.3390/nu15224727)
Supplement: Supplementary file 1 [file nutrients-15-04727-s001.zip › nutrients-2656932-supplementary.pdf]

Supplementary Tables

Table S1: modified GLIM criteria

PHENOTYPIC CRITERIA
Weight loss > 5% in the last 6 months or > 10% beyond 6 months
Low BMI: < 20 if age < 70 years or < 22 if age > 70 years
ETIOLOGIC CRITERIA
Reduced food intake or assimilation
Inflammation: acute disease or chronic disease

Table S2: Variables included in the computation of the frailty index

**Variables included in the computation of the frailty index**
Istitutionalized
Hypertension
Diabetes
Dyslipidemia
Coronary artery disease
Atrial fibrillation
Heart Failure
Pulmonary Embolism/Deep venous thrombosis
Asthma
Stroke/TIA
Parkinson
Alzheimer
Other dementia
Other neurologic disorders
Arthrosis
Depression
Hepatic disease
Anemia (chronic)
myelodysplastic syndrome/myeloproliferative syndrome
Solid tumor
taking psychoactive drugs
polytherapy
Chronic ACB score elevated
Any_ACB elevated during hospital stay
Any_Na_high or low
Any_SO2/FiO2 moderately altered
Any_K_low or high
Any_ANEMIA_of moderate degree
Any glycemia > 200 mg/dl

Table S3: evaluations performed during the study

	Hospital admission	Hospital stay	One month after hospital discharge
MUST	x		
GLIM criteria of malnutrition	x		x
MNA-SF			x
4AT	x	x	
Height			x
Weight			x
Calf circumference			x
Waist circumference			x
SARC-F			x
SPPB			x
6MWT			x

**LEGEND**	
MUST	Malnutrition Universal Screening Tool
GLIM	Modified Global Leadership Initiative on Malnutrition
MNA-SF	Mini-Nutritional Assessment Short Form
SARC-F	Strength, Assistance with walking, Rising from a chair, Climbing stairs, and Falls
SPPB	Short Physical Performance Battery
6MWT	6-minute walk test
